# Ethosuximide-loaded bismuth ferrite nanoparticles as a potential drug delivery system for the treatment of epilepsy disease

**DOI:** 10.1371/journal.pone.0305335

**Published:** 2024-09-23

**Authors:** Yeliz Guldorum, Musa Ayran, Burcak Bulut, Sule Ilgar, Songul Ulag, Zehra Kanli, Banu Aydin, Rezzan Gulhan, Tuba Bedir, Oguzhan Gunduz, Roger J. Narayan

**Affiliations:** 1 Department of Biomedical Engineering, Electrical and Electronics Faculty, Yıldız Technical University, Istanbul, Turkey; 2 Center for Nanotechnology & Biomaterials Application and Research (NBUAM), Marmara University, Istanbul, Turkey; 3 Institute of Pure and Applied Sciences, Metallurgical and Materials Engineering, Marmara University, Istanbul, Turkey; 4 Department of Bioengineering, Faculty of Chemical and Metallurgical Engineering, Yildiz Technical University, Istanbul, Turkey; 5 Department of Metallurgical and Materials Engineering, Faculty of Technology, Marmara University, Istanbul, Turkey; 6 Department of Biophysics, School of Medicine, Marmara University, Istanbul, Turkey; 7 Department of Medical Pharmacology, School of Medicine, Marmara University, Istanbul, Turkey; 8 Epilepsy Research and Implementation Center, Marmara University, Istanbul, Turkey; 9 Joint Department of Biomedical Engineering, University of North Carolina, Chapel Hill, NC, United States of America; Univerzitet Singidunum, SERBIA

## Abstract

Encapsulating antiepileptic drugs (AEDs), including ethosuximide (Etho), into nanoparticles shows promise in treating epilepsy. Nanomedicine may be the most significant contributor to addressing this issue. It presents several advantages compared to traditional drug delivery methods and is currently a prominent area of focus in cancer research. Incorporating Etho into bismuth ferrite (BFO) nanoparticles within diverse controlled drug delivery systems is explored to enhance drug efficacy. This approach is primarily desired to aid in targeted drug delivery to the brain’s deepest regions while limiting transplacental permeability, reducing fetal exposure, and mitigating associated adverse effects. In this investigation, we explored Etho, an antiepileptic drug commonly employed for treating absence seizures, as the active ingredient in BFO nanoparticles at varying concentrations (10 and 15 mg). Characterization of the drug-containing BFO nanoparticles involved scanning electron microscopy (SEM) and elemental analysis. The thermal properties of the drug-containing BFO nanoparticles were evaluated via differential scanning calorimetry (DSC) analysis. Cytotoxicity evaluations using the MTT assay were conducted on all nanoparticles, and human neuroblastoma cell line cultures (SH-SY5Y) were treated with each particle over multiple time intervals. Cell viability remained at 135% after 7 days when exposed to 15 mg of Etho in BFO nanoparticles. Additionally, *in vitro* drug release kinetics for Etho revealed sustained release lasting up to 5 hours with a drug concentration of 15 mg.

## Introduction

Epilepsy, a widely prevalent neurological disorder worldwide, is primarily characterized by abnormal electrical activity in various brain regions. Neurodegeneration often results from excessive Ca^2+^ influx into neurons, causing cell death. Despite the availability of numerous antiseizure drugs, a substantial number of patients remain unresponsive to treatment, experiencing refractory epilepsy [1]. The fundamental approach in the treatment of epilepsy is to control the patient’s seizures by using the antiepileptic drug [2]. However, despite this medication successfully reducing or eliminating epileptic seizures in 70% of patients, it is ineffective in the remaining patients due to drug resistance [3]. In patients whose epileptic crises are uncontrollable by pharmaceuticals, surgical treatment is an alternative option [4]. Conventional treatments still have limitations, as most antiepileptic drugs cannot cross the blood-brain barrier [5]. Controlled drug delivery systems are considered promising candidates for addressing the limitations of conventional approaches.

The blood-brain barrier (BBB) serves as both a physical and metabolic barrier, regulating the exchange of xenobiotics and nutrients across the BBB while safeguarding the brain’s microenvironment [6, 7]. In this context, two primary hypotheses regarding antiseizure medication (ASM) resistance are prominent: the target and transporter theories. The former posits that molecules targeted by ASMs undergo modifications that ultimately diminish the therapeutic effectiveness of ASM. The latter hypothesis asserts that the malfunctioning of multidrug transporters leads to a reduction in the effective concentration of ASM within the brain [8, 9]. In light of this, nanoparticles represent a promising alternative for overcoming the BBB and achieving therapeutic ASM levels [1].

Moreover, biodegradable nanoparticles are emerging as promising therapeutic approaches for managing epilepsy. These nanoparticles can traverse BBB, enhance brain-specific targeting, reduce side effects, and provide sustained drug delivery. Furthermore, biodegradable nanomaterials are able to naturally degrade into non-toxic byproducts within the body. They can also be engineered to degrade specifically at the target site while maintaining stability in off-target areas [10, 11].

Etho is a drug that readily dissolves in water and is considered a potential inhibitor of T-type Ca^2+^ channels [12]. Apart from the mentioned Ca^2+^ channel-blocking and neurochemical effects attributed to Etho, an additional localized action of Etho within the brainstem has been suggested [13]. Metallic nanoparticles have gained attention in epilepsy research due to their dense surface functionalization, small size-to-volume ratio, stability, and easy detectability. These properties offer potential benefits not only for treatment but also for diagnosis [14]. The inclusion of bismuth ferrite (BFO) nanoparticles in composites has a profound impact on cell behavior. BFO, with its ferroelectric and piezoelectric properties, generates an electric field within the composite. This electric field enhances cell attachment, influences gene and protein expression, and modifies the mechanical and chemical properties of the biopolymer. Incorporating BFO nanoparticles can also lead to an augmented porosity in the structures, offering potential advantages in areas like such as tissue engineering and drug delivery [15, 16]. Due to their extensive surface area and nanoscale dimensions, nanomaterials find wide-ranging applications in drug delivery [17, 18].

Conventional medicines often face a significant barrier in reaching the brain due to the absence of drug-specific transport systems across the BBB. However, drug-loaded nanoparticle technology presents a promising solution to circumvent this challenge. By encapsulating drugs within nanoparticles, the limitations posed by the BBB can be overcome, enabling more effective delivery of medications to the brain. This approach may enhance drug delivery to the brain for the treatment of epilepsy and other neurological disorders. To our best understanding, this study represents the initial investigation for the treatment of epilepsy, showcasing the impact of Etho on SH-SY5Y cells, along with its integration into BFO nanoparticles. This research provides novel insights into the effects of Etho in this particular cell line and highlights its possible collaboration with BFO nanoparticles. The aim of this study, beyond the characterization of Etho-reinforced BFO nanoparticles, is to investigate the effects of their antitoxic effects on neural cells after drug release studies. Furthermore, the characterization studies contribute to a better understanding of the properties of Etho and its combination with BFO nanoparticles, which may advance efforts to use nanostructured materials for medical applications.

## Materials and methods

### Materials

Bismuth (III) nitrate pentahydrate [Bi(NO_3_)_3_·5H_2_O] was purchased from Merck (Darmstadt, Germany). Iron (III) nitrate nonahydrate [Fe(NO_3_)_3_.9H_2_O] was purchased from Merck (Darmstadt, Germany). Nitric acid (65%) was provided by Merck (Darmstadt, Germany). Ethosuximide was bought from Sigma Aldrich, USA.

### Etho-reinforced BFO synthesis with co-precipitation method

BFO nanoparticles were synthesized using the co-precipitation method with iron (III) nitrate [Fe(NO_3_)_3_·9H_2_O], nitric acid (HNO_3_), bismuth (III) nitrate [Bi(NO_3_)_3_·5H_2_O], and ammonia (NH_4_OH). Two different beakers were used to prepare Bi(NO_3_)_3_ and Fe(NO_3_)_3_ solutions. Firstly, 1.86 g of Bi(NO_3_)_3_ was weighed and added to 10 mL of nitric acid. Then, 2.58 g of Fe(NO_3_)_3_ was weighed and added to 10 mL of distilled water, stirring at 300 rpm for 15 minutes using a magnetic stirrer without applying heat. Once the two solutions were dissolved entirely, they were combined in one beaker, and the drug mixture was added at this stage. The drug mixture was stirred in a magnetic stirrer at 300 rpm without heat for 40 minutes. Two drug solutions were prepared, one with Etho 10 mg (BFO_10 Etho) and the other with 15 mg (BFO_15 Etho). After observing the complete dissolution of the drug, pH adjustments were made using ammonia in a fume hood. Approximately 40 mL of ammonia solution was slowly added to the solution using a glass pipette, and the precipitates formed during the addition were stirred with a spoon after each addition. Once the pH reached 10.20, the precipitated BFO was purified from ammonia through six washes with distilled water and filter paper to remove toxic substances. The BFO collected on the filter paper was placed in an oven at 100°C for 24 hours to evaporate excess water. The resulting BFO was crushed into powder form using a mortar and pestle and stored until the subsequent use.

### Fourier transform infrared spectroscopy (FTIR)

An FTIR Spectrometer (Jasco, FTIR 4700) emerged as the tool of choice to investigate the intricate chemical composition of the nanoparticles. This analytical powerhouse operated across a comprehensive scanning range, spanning from 400 to 4000 cm⁻^1^, ensuring an encompassing analysis of molecular vibrations and bonds. The achieved resolution of 4 cm⁻^1^ further heightened the precision of this spectral inspection.

### Scanning electron microscope (SEM) analysis

After coating the surfaces of the nanoparticles with gold, SEM was employed to conduct the morphological analysis using the SEM (EVA, MA 10) instrument from ZEISS in Pleasanton, CA, USA. The Olympus AnalySIS imaging software (Waltham, MA, USA) was utilized to analyze the average particle size and distribution of nanoparticles. Energy dispersive spectroscopy (EDS) was employed to analyze BFO and Etho-reinforced BFO nanoparticles. The powders of nanoparticles were coated with the Au element for 60 seconds using Quorum SC7620.

### Differential scanning calorimeter (DSC) analysis

Thermal characterization was conducted on both BFO and BFO nanoparticles reinforced with Etho. This analysis was performed using a Shimadzu DSC-60 Plus machine through DSC. The temperature range for the study spanned from 25°C to 600°C, and the heating rate was set at 10°C/min.

### X-ray diffraction (XRD) analysis

The crystal structure of BFO powders was investigated using an XRD device with a Cu source (λ = 1.54060 A°). The acquisition parameters of the XRD system were optimized at 40 kV and 30 mA, while the scan range covered 10–80° with a scan speed of 0.48 (°/min) and a preset time of 2 seconds. The Debye-Scherrer formula was utilized to determine the crystallite size by evaluating the full width at half maximum.

### Dynamic light scattering (DLS), zeta potential analysis

The assessment of polydispersity index (PDI), particle size, and zeta potential of the nanoparticles was conducted utilizing a NanoZS Instrument manufactured by Malvern Instruments (Malvern, United Kingdom). The measurements were performed at a temperature of 25°C, wherein 1 ml of the suspended nanoparticle solution, dispersed in ethanol, was introduced into the sample chamber of the device. Each formulation underwent three successive measurements to ensure the consistency and reliability of the data.

### *In vitro* drug release studies

The linear calibration curves for Etho were established, employing five distinct concentrations of Etho (0.2, 0.4, 0.6, 0.8, and 1 mg/mL). To ascertain the absorbance values of the Etho, a UV-Vis spectrophotometer was employed, operating at a wavelength of 203 nm specifically for Etho. The nanoparticles incorporating Etho were sectioned into smaller fragments (10 mm × 10 mm). These fragments were then immersed in 1 ml of phosphate-buffered saline (PBS) and subjected to incubation alongside the nanoparticles. Maintained at 37°C, the incubation process was carried out using a thermal shaker (BIOSAN TS-100) operating at 400 rpm. To investigate the release kinetics of the antiepileptic drug Etho, distinct time intervals spanning up to 300 minutes were employed. The PBS-loaded nanoparticles collected at specific time points were analyzed using a UV-Vis spectrophotometer (Shimadzu, 190–220 nm). Following each measurement, 1 ml of fresh PBS was introduced to the existing samples in preparation for subsequent assessments.

### Cytotoxicity assay

For the culture of the human neuroblastoma cell line SH-SY5Y (CRL-2266™, ATCC), 25 cm^2^ flasks were utilized; the cells were supplemented with 10% (v/v) inactivated fetal calf serum (FCS) (Thermo Fisher Scientific), 2 mM L-glutamine (Sigma), 100 IU/ml penicillin, and streptomycin in Dulbecco’s Modified Eagles Medium (DMEM, Gibco) at a cell density of 10^6^ cells/ml. The cells were maintained within a humidified atmosphere with 5% CO_2_ at 37˚C. To ensure optimal growth, the culture medium was replaced every 2 days. In addition, the cell cultures were split twice a week. Cell viability was closely monitored, and when the cells reached approximately 80% confluence, they were subjected to trypsinization using trypsin-EDTA from Gibco. Following this process, the cells were washed and evaluated for viability. After an overnight recovery, the culture medium was replaced with a fresh, nutrient-rich medium, promoting continued growth and experimentation.

Regarding the purpose of DAPI staining, SH-SY5Y cells cultured, as explained previously, were subjected to Etho treatment for the specified durations. Following the treatment, the cells were cleansed with PBS and subsequently fixed using ice-cold acetone for 30 minutes. Thereafter, the cells underwent two additional PBS washes and were subsequently stained with DAPI (300 nM) in a light-free environment for 10 minutes. Subsequently, the cells were observed under a fluorescent microscope, and the visual data were captured using a Leica DMI 6000B fluorescence microscope (Wetzlar, Germany).

## Results and discussion

**Fig 1:**
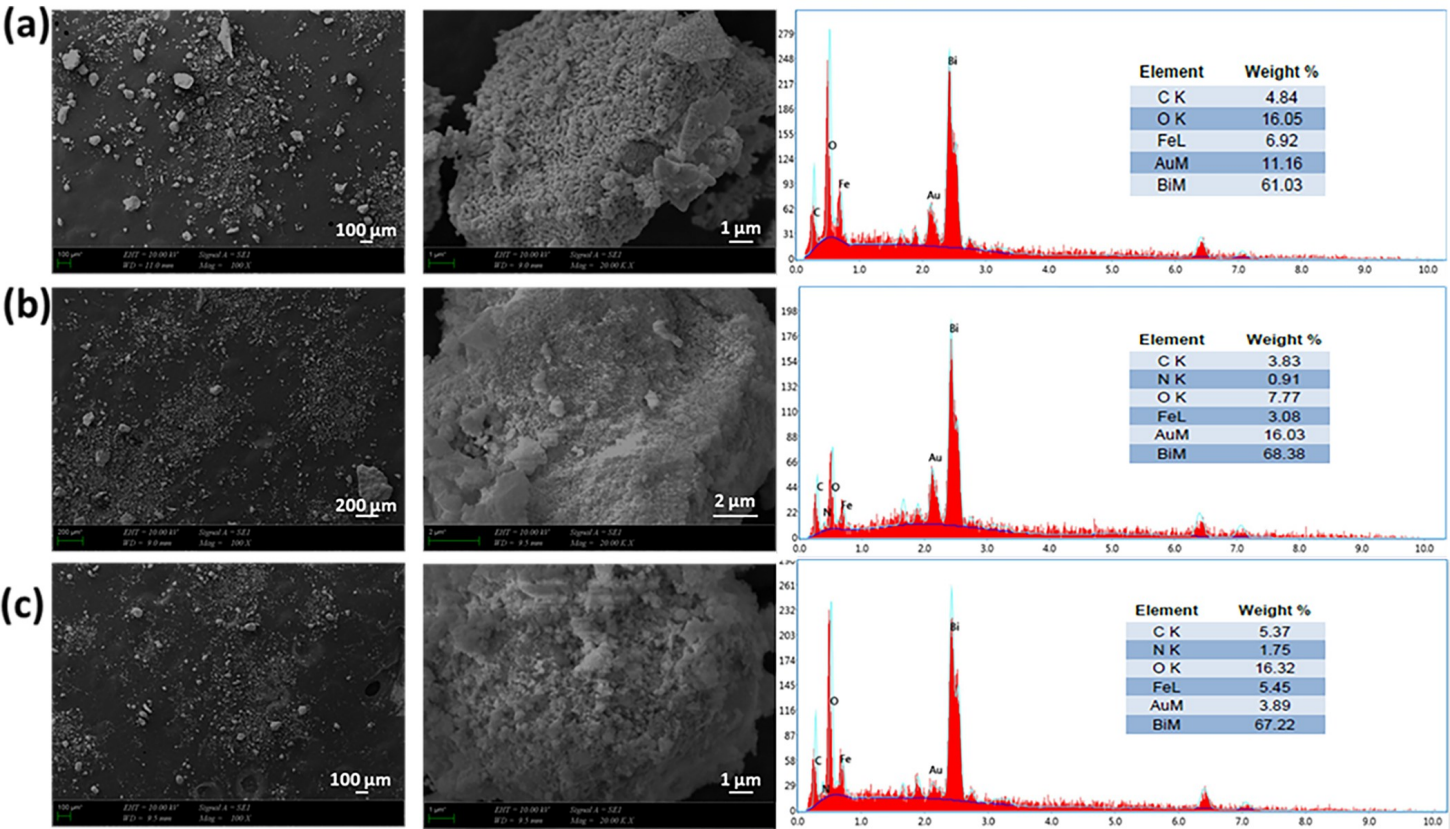
Morphological characterization and elemental analysis of BFO (a), BFO with Etho 10 mg (BFO_10 Etho) (b), and BFO with Etho 15 mg (BFO_15 Etho) (c) using SEM.

An investigation was conducted to examine how the presence of Etho affects the morphology of BFO nanoparticles, and it was subsequently observed that increasing the concentration of Etho led to changes in the BFO nanoparticles. Fig 1 depicts SEM images of BFO nanoparticles and Etho-reinforced BFO nanoparticles and their elemental analysis. The energy dispersive spectra obtained from SEM-EDS analysis for the nanoparticles provide clear evidence that the nanoparticles were synthesized using the aforementioned method. With regard to SEM visual inspection, variations in the homogeneous distribution of nanoparticle sizes among the different groups become apparent. Particularly, in the case of BFO images, the presence of larger nanoparticles is observed. However, as the drug is introduced, there seems to be a reduction in the abundance of these larger nanoparticles, suggesting a trend toward a more homogeneous behavior. In terms of their small particle size, nanoparticles exhibit the potential to enhance drug stability, prolong therapeutic efficacy, minimize metabolic degradation, and enhance cellular uptake [19]. In the literature, nanoparticles are defined as solid colloidal particles falling within the size range of 10 nm to <1000 nm. However, for applications in nanomedicine, a preferred size range is typically less than 200 nm [20, 21]. In this specific case, the sizes of nanoparticles appear aggregated, making precise measurements challenging; however, it is anticipated that the sizes fall within the preferred range. Owing to the intrinsic properties of BFO, the nanoparticles exhibit a propensity to coalesce and form aggregates; this phenomenon is attributed to factors such as their magnetic dipolar interactions, extensive surface area, and van der Waals forces [22]. Given this characteristic, additional functionalization procedures applied to BFO nanoparticles may prove insufficient in mitigating agglomeration. Nanoparticles synthesized at elevated temperatures tend to promote the creation of more uniform agglomerates, which are characterized by mechanically robust and compact structures [23]. Despite employing a low-temperature process in our investigation, coalescence phenomena were still detected. It is important to note that further analysis is required to provide a detailed examination of nanoparticle size to substantiate this observation. In addition, the images reveal nanoparticle agglomeration, which is more pronounced with the introduction of Etho. Importantly, it becomes evident that the increase of Etho exacerbates the degree of agglomeration within the nanoparticle structures.

**Fig 2:**
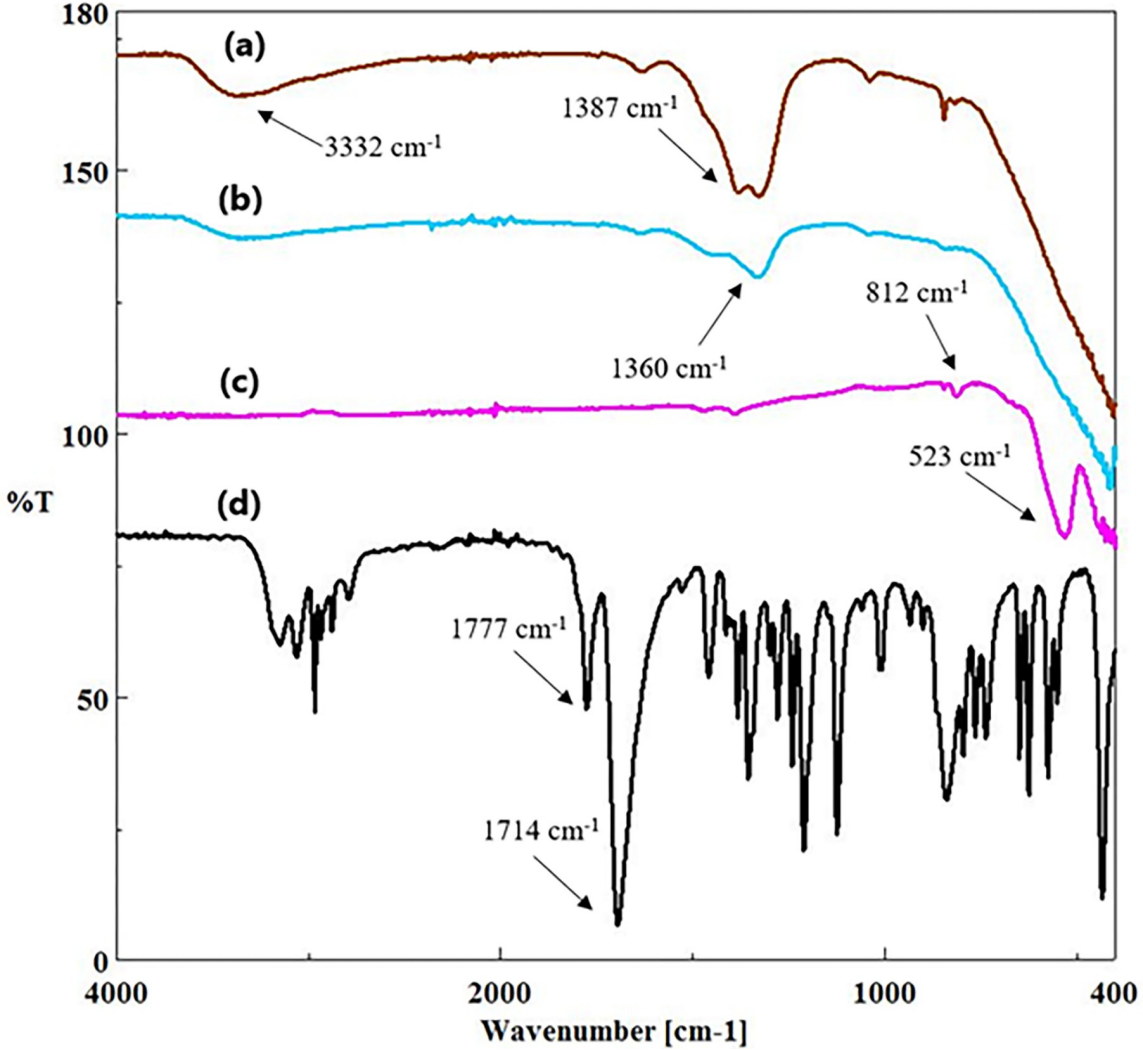
Physicochemical characterization of BFO_15 Etho (a), BFO_10 Etho (b), BFO (c), and Etho (d) using FTIR.

In Fig 2, we present the FTIR spectra of both BFO nanoparticles and Etho-reinforced BFO nanoparticles. The BFO nanoparticle spectrum exhibited distinct absorption peaks at ~523 cm^−1^ and ~451 cm^−1^, representing characteristic ferrite features associated with metallic ions [24]. Furthermore, the peak at ~812 cm^−1^ was attributed to NO_3_^−^ variation [25]. Within the range of 450–525 cm^−1^, bands indicative of ferrite were observed, corresponding to metallic ions. These bands, which are related to the bending vibrations and stretching of Fe-O bonds, suggest the presence of [FeO_6_] octahedra in perovskites [16, 26], in which metal ions are bonded to these structures. However, these peaks disappeared upon the introduction of Etho drugs into the BFO nanoparticles, as seen in Fig 2A and 2B. The significant C = O stretching vibration peaks observed at 1777 cm^-1^ and 1714 cm^-1^ in the FTIR spectrum of pure Etho were notably absent in the spectrum of Etho after its incorporation with BFO nanoparticles [27, 28]. This disappearance of strong C = O peaks indicates that the encapsulation process effectively shielded the functional groups of Etho. In addition, within the FTIR spectra of Etho-reinforced BFO nanoparticles, the characteristic peaks associated with Etho, such as those at 1387 cm^-1^ and 1360 cm^-1^ (related to CH_3_ vibrations) and 3332 cm^-1^ (related to N-H vibrations), appear to be present, albeit with reduced intensity compared to pure Etho. The peak at 1387 cm^-1^ can be attributed to the CH_3_ wagging vibration of the methylene (-CH_2_^-^) group in Etho. Methyl groups are commonly known as electron-donating substituents within aromatic ring systems [28]. In conclusion, the disappearance of some of the characteristic peaks of Etho and the reduced intensity of others upon its incorporation into BFO nanoparticles suggest the possibility of partial encapsulation.

**Fig 3:**
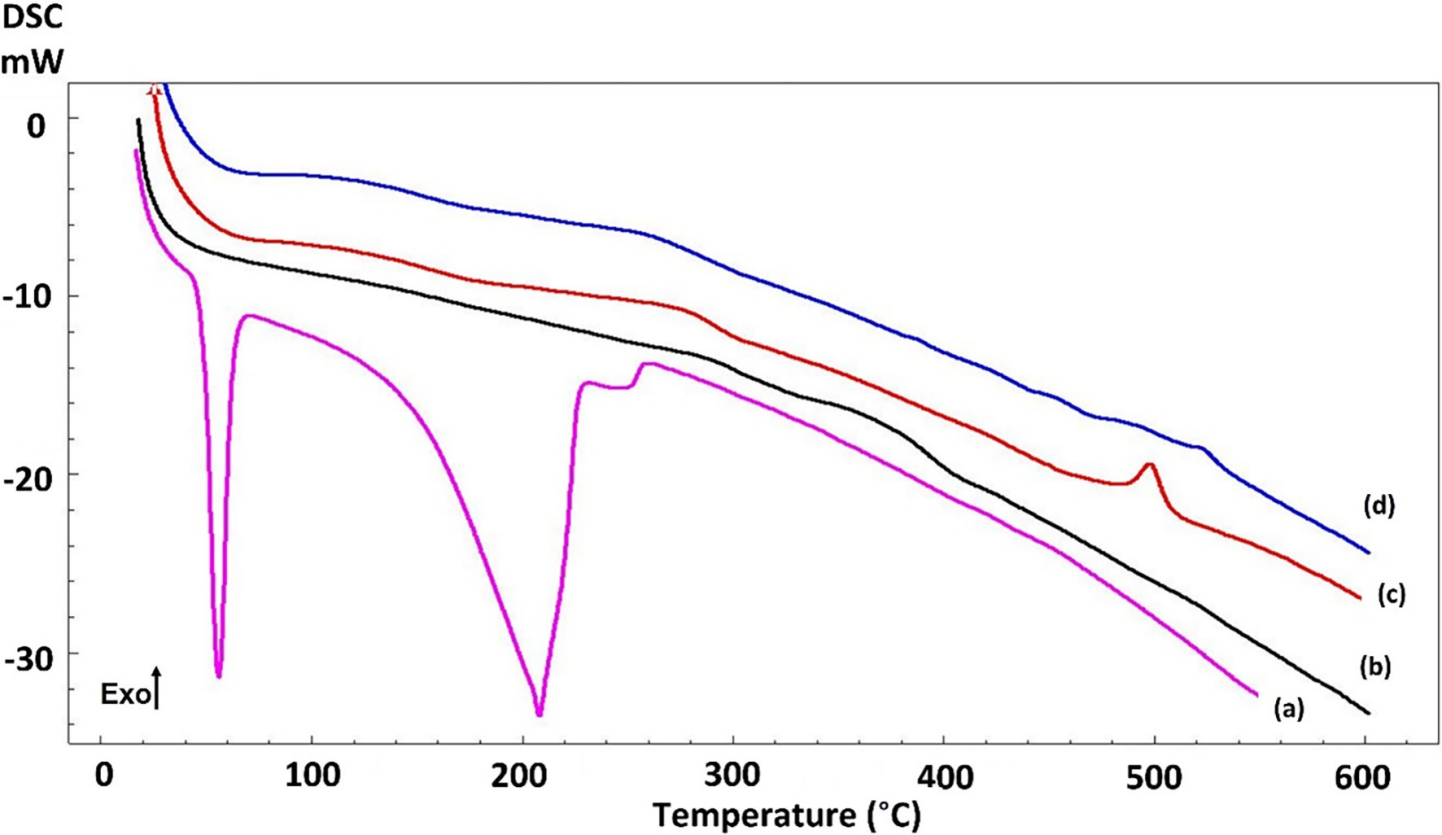
DSC analysis of Etho (a), BFO (b), BFO_10 Etho (c), and BFO_15 Etho (d).

The thermal characteristics of both Etho nanoparticles, BFO nanoparticles, and nanoparticles reinforced with Etho were examined, and the corresponding data is illustrated in Fig 3. In the DSC thermogram of pure Etho, an endothermic peak at 47.8°C was observed, primarily attributed to its phase transition. This finding aligns with previous studies on Etho, where it was observed that Etho exhibits its phase transition at this same temperature range [29]. Furthermore, in pure Etho, a broad endothermic peak is observed at around 215°C, which may represent the melting temperature of the succinimide group. Specifically, this melting point might indicate the temperature at which the atoms and molecules within the succinimide group’s crystal structure release their energy. BFO nanoparticles do not typically exhibit sharp, well-defined peaks in a DSC graph because it does not undergo a distinct phase transition, such as melting or crystallization, over a narrow temperature range under standard conditions [15]. In the scientific literature, it has been reported that BFO undergoes a phase transformation in the 755–817°C temperature range, shifting from a rhombohedral structure to a cubic phase [30]. Including Etho in BFO nanoparticles did not induce any changes in thermal characteristics, even with an increased Etho concentration, as evidenced by the absence of endothermic peaks in Etho-reinforced BFO nanoparticles. It is possible that the concentration of Etho added to the BFO nanoparticles was not high enough to induce noticeable changes in the thermal properties.

**Fig 4:**
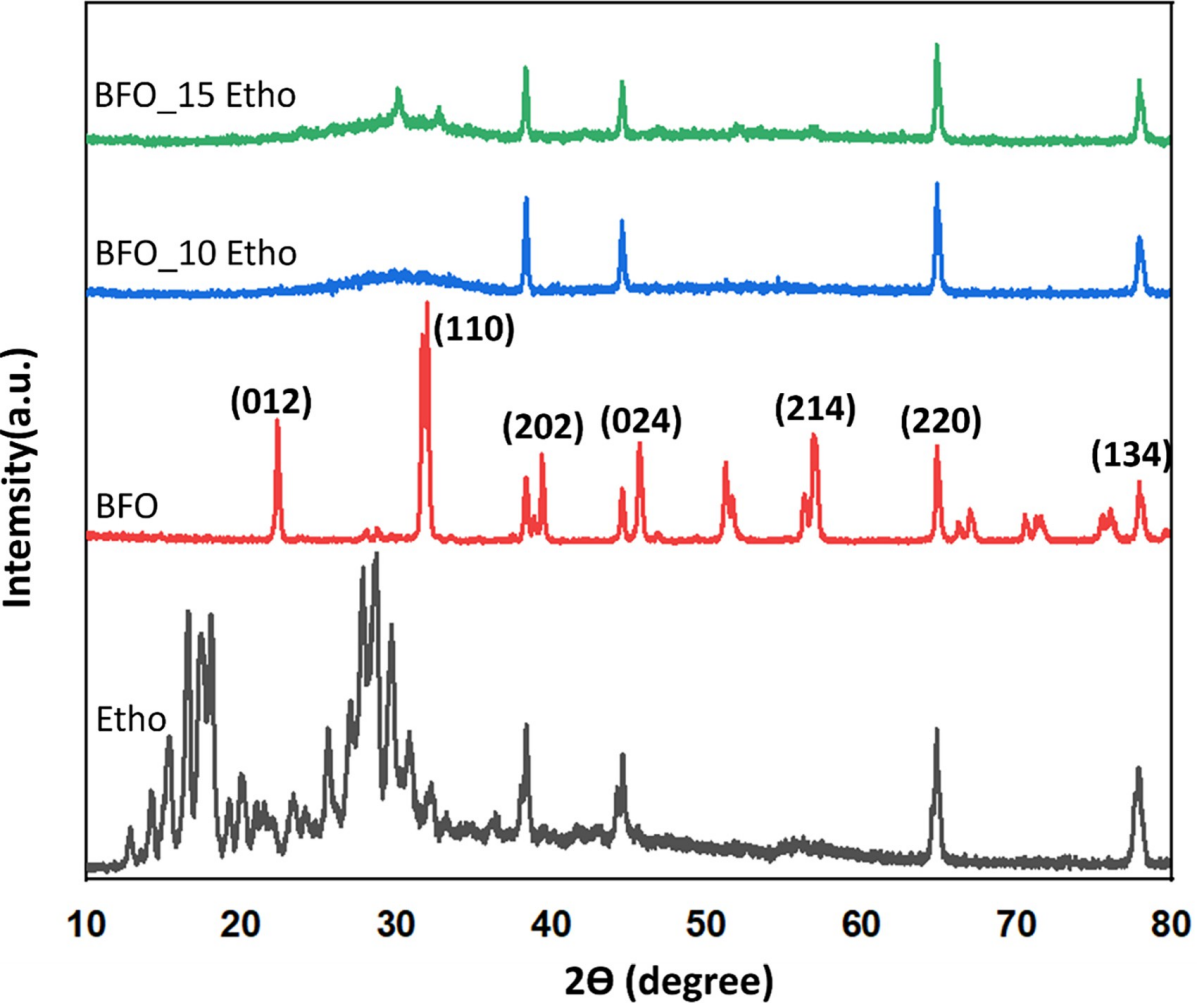
XRD analysis for crystallographic characterization of Etho, BFO, BFO_10 Etho, and BFO_15 Etho particles.

Fig 4 presents XRD patterns of nanoparticles to evaluate their crystalline phases. It includes patterns for Etho, BFO, BFO_10 Etho, and BFO_15 Etho. Only pure BFO nanoparticles of the diffraction peaks have been associated with different (*hkl*) planes within the hexagonal crystal system. The presence of sharp and prominent peaks in the X-ray diffraction patterns confirms the outstanding crystalline quality of the BFO nanoparticles synthesized using co-precipitation, while the low-intensity peaks indicate the presence of secondary phases [25]. The prominent diffraction peaks were observed at 2θ angles of 22.4, 27.5, 31.7, 32, 39.4, 45.7, 51.2, 57, and 66°. These peaks closely correspond to the XRD pattern of a single-phase BFO material with a distorted perovskite structure in the rhombohedral R3c crystallographic group [31]. Additionally, the hexagonal crystal structure of BFO was verified through the distinct splitting of peaks at (104) and (110). The presence of sharp and intense XRD peaks indicates the excellent crystalline quality of the synthesized BFO nanoparticles. However, these peaks disappeared with the addition of Etho into BFO nanoparticles. This is attributed to the binding of Etho to the surface or within the BFO nanoparticles, potentially causing disruptions in the crystalline structure of the nanoparticles. The addition of Etho in BFO nanoparticles can lead to the formation of aggregates. Since these aggregates have a larger volume, it can result in a less uniform crystalline structure of the nanoparticles. Consequently, this leads to lower intensities of XRD peaks. In the case of BFO_15 Etho, a peak at 2θ: 30 degrees was observed; this peak may be attributed to agglomeration. The addition of Etho increased disorder within the BFO crystal structure, potentially leading to a transformation towards a more amorphous structure.

**Fig 5:**
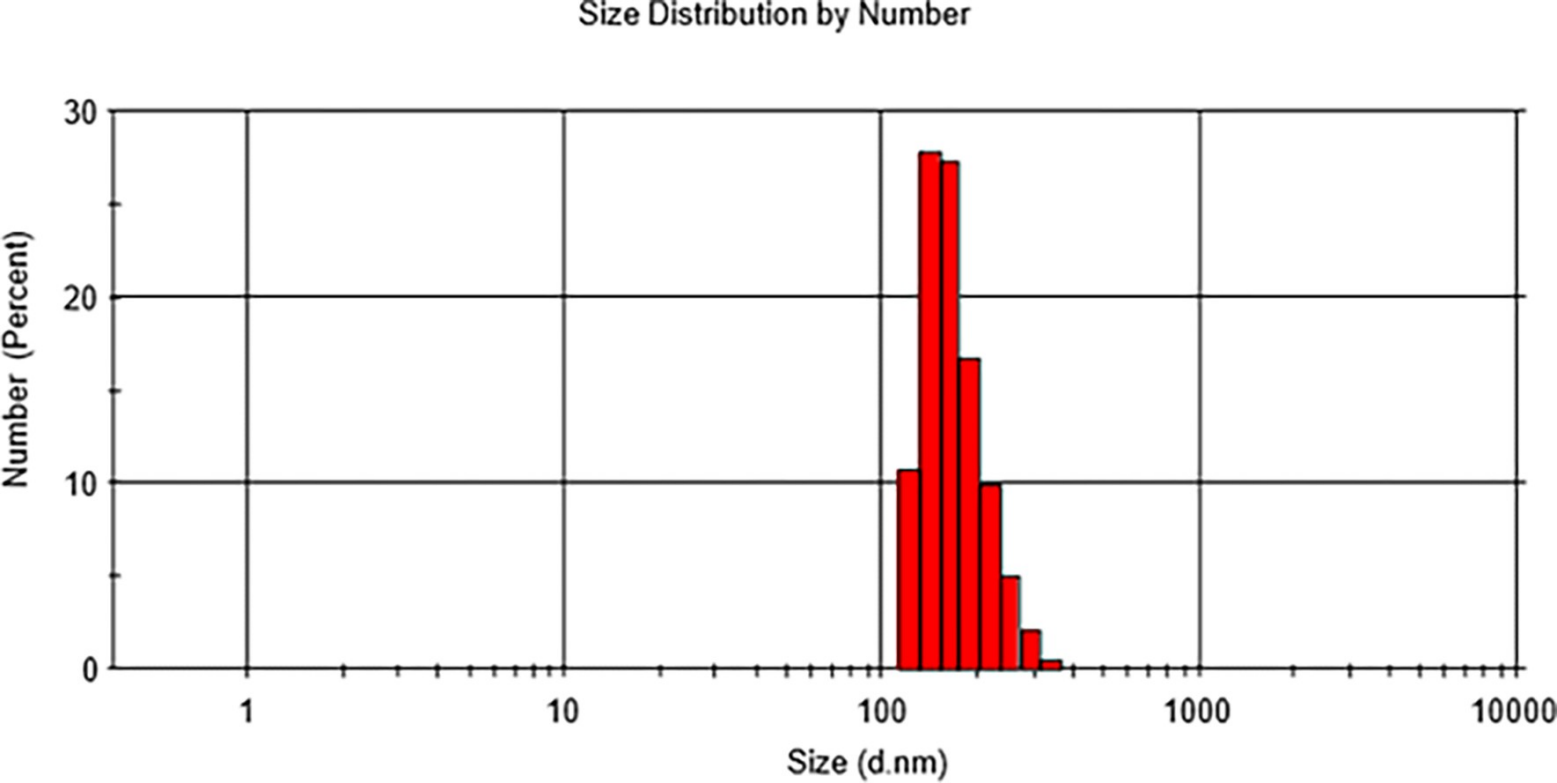
Size distribution of BFO nanoparticles as determined through DLS.

The zeta potential and particle size of the BFO nanoparticles were assessed using the DLS technique. The sample underwent dissolution in a 99% ethanol solution and subsequent treatment with an ultrasonicator for a duration of 1 minute. The resulting average particle size was measured as 171.7 nm, with a corresponding zeta potential recorded as -18 mV. The zeta potential value of BFO nanoparticles aligns with previous reports [32].The observed PDI values stood at 0.05. The size distribution of BFO nanoparticles (shown in Fig 5) fell within a narrow range (roughly 20–350 nm), suggesting the creation of particles that are relatively uniform in size. The PDI value also serves as an indicator of the uniformity or homogeneity of the BFO particles. The average size of BFO nanoparticles was determined to be less than 200 nm, suggesting their capability to effectively access endothelial cells. Futhermore, nanoparticles with sizes below 200 nm exhibit enhanced blood circulation, which is associated with prolonged contact time between the drug and the BBB [33].

**Fig 6:**
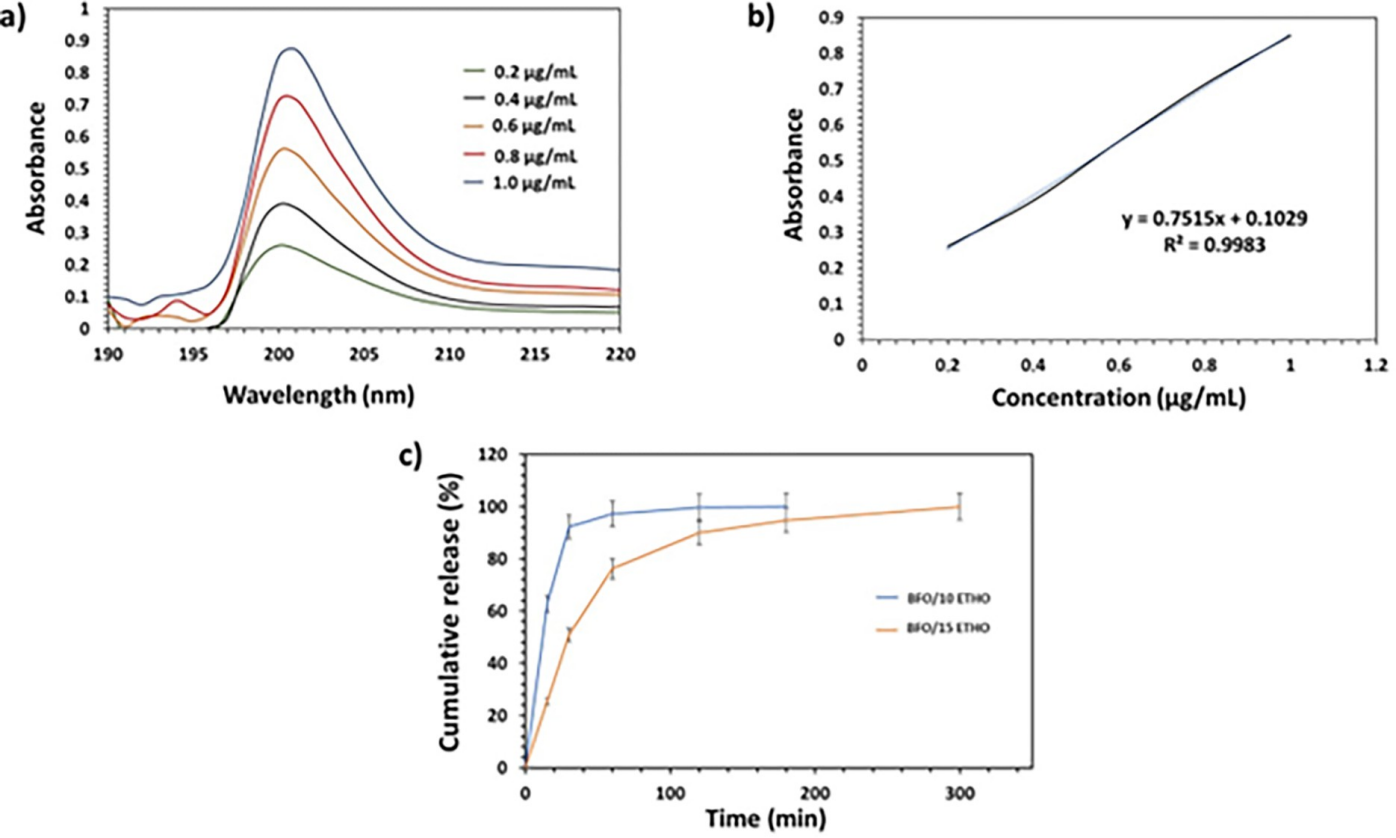
Comprehensive analysis of Etho release from BFO nanoparticles: calibration curve (a), absorbance profile (b), and cumulative drug release kinetics (c) under *in vitro* conditions.

While antiepileptic drugs (AEDs) are frequently administered via the oral route and intravenous route, these methods may not always be associated with the desired efficacy [34]. Among different administration routes, the oral route stands out as the most prevalent administration route. The sublingual route, which involves the placement of medication under the tongue, has emerged as a promising alternative to the oral administration method for drug delivery [35]. We have considered encapsulating AEDs within sustained nano-delivery systems. This approach can support the development of novel drug delivery strategies, including the encapsulation of drugs within metallic nanoparticles. Encapsulated Etho in BFO nanoparticles are designed to undergo rapid disintegration or dissolution upon contact with a small volume of saliva, thereby facilitating swift drug absorption through the mucosa. Consequently, we conducted a modified dissolving technique in PBS medium using a thermal shaker. The obtained results are presented in Fig 6. Fig 6A illustrates the calibration curve, which was established using five distinct solutions. Fig 6B displays the measured absorbance of Etho at 203 nm, while Fig 6C presents the cumulative release profile of Etho from BFO nanoparticles. According to the cumulative release profile, within the initial 15 minutes, the release percentage reached 28.50%. After 30 minutes, the release percentage increased to 51.78%. Subsequently, approximately 73.8% of the BFO_15 Etho was released after one hour. At a lower Etho concentration, rapid release kinetics were observed in BFO_10 Etho. BFO_10 Etho exhibited the burst release (61.20%) within the initial 15 minutes, 82.5% release within the first half-hour, and 93% release within the initial hour. The complete release was achieved within 3 hours, reaching equilibrium. These results revealed that BFO_15 Etho displayed a more prolonged and sustainable release profile. Notably, the concentration of Etho significantly influences the release kinetics, leading to a more controlled and prolonged release as the Etho concentration increases. Furthermore, BFO_15 Etho demonstrates a remarkable potential for drug delivery applications, particularly in scenarios where achieving sustained release with minimal initial burst release is a priority. This phenomenon also can be attributed to the encapsulation process, which warrants further explanation. Moreover, the development of Etho-reinforced BFO nanoparticles can be a promising option for the treatment of epilepsy. Additionally, the potential of nanoparticles to cross the blood-brain barrier has made them a central research focus in brain drug delivery [36]. Lastly, these nanoparticles can be designed to deliver drugs to the brain in a controlled and targeted manner, which can help improve treatment efficacy and safety.

**Fig 7:**
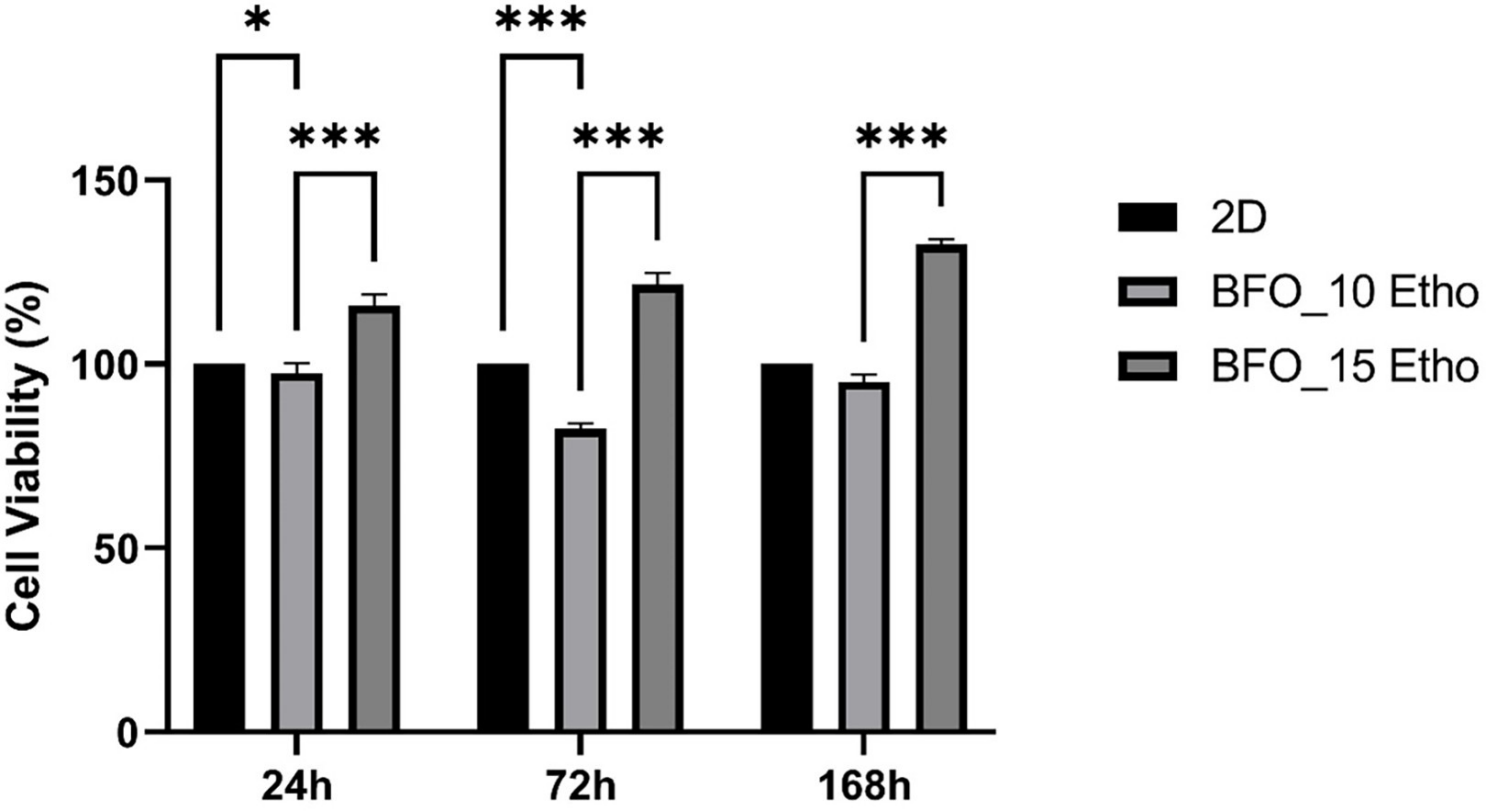
The MTT assay was employed to evaluate the cytotoxic effects of Etho-reinforced BFO nanoparticles on SH-SY5Y cells, and the results were analyzed on 1, 3, and 7 days. The statistical analysis, including applying the two-way ANOVA test, was conducted utilizing GraphPad Prism software (*p < 0.05, **p < 0.01).

In order to evaluate cell viability, the cytotoxicity of Etho combination with BFO was examined using the MTT assay over several time periods, as can be seen in Fig 7. The findings revealed that concentrations of Etho exceeding 10mg exhibited considerable cytotoxic effects. When 15 mg of Etho-reinforced BFO nanoparticles were introduced, cell viability reached 115% on the first day, increased to 121% on the third day, and further improved to 135% on the seventh day. In contrast, introducing 10 mg Etho-reinforced nanoparticles resulted in 98% cell viability on the first day, declining to 80% on the third day, and subsequently recovering to 95% on the seventh day. BFO_15 ETHO exhibited consistently higher bioavailability compared to BFO_10 ETHO across all of the observed time points. These findings suggest a positive correlation between the Etho concentration and cell viability. Within the spectrum of treatment modalities, a notably promising strategy involves the precise administration of drugs directly to the epileptic focus region [37]. This approach aims to achieve a substantial drug concentration at the intended target, all the while mitigating potential toxicity on the surrounding neural network. In line with this strategy, our findings indicate that employing a substantial concentration of Etho within BFO nanoparticles does not lead to toxicity. Furthermore, by achieving targeted and prolonged drug delivery, this treatment reduces drug-related side effects and enhances patient compliance through less frequent dosing [20]. From this perspective, we can suggest that Etho_15 BFO may exhibit lower toxicity due to its more controlled and prolonged release profile.

**Fig 8:**
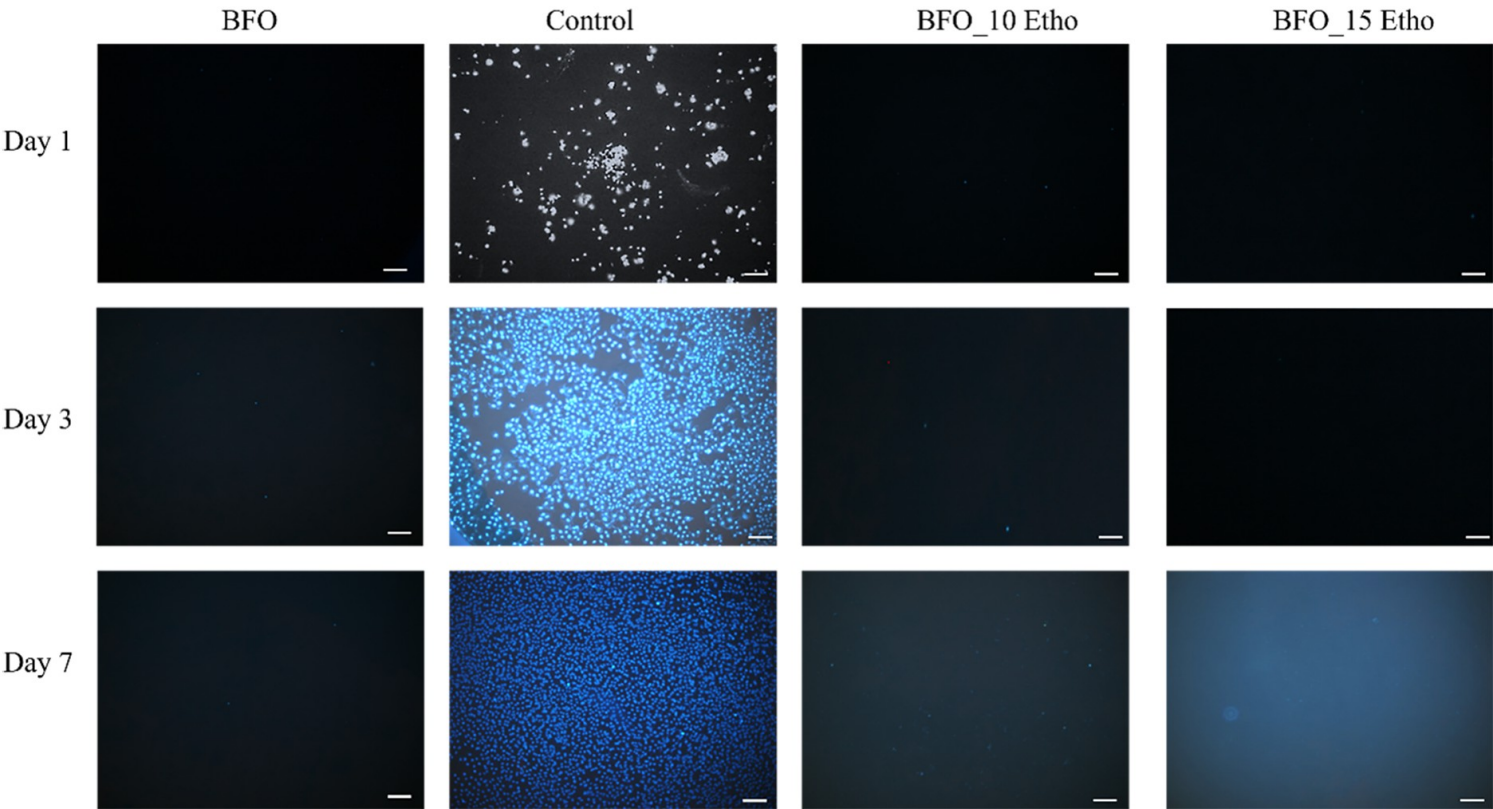
The fluorescence images of the particles after 1, 3, and 7 days of incubation.

In Fig 8, fluorescence images depict the adhesion of SH-SY5Y cells to the Etho-reinforced BFO nanoparticles at various time intervals. DAPI staining was employed to quantify the nuclei count and evaluate the overall cellular morphology [38]. In DAPI images depicting cell adhesion, low levels of cell adhesion are observed in all groups except the control. Based on these results, it cannot be definitively concluded that there is substantial cell adhesion. When analyzing the correlation between Etho-reinforced BFO nanoparticles, it becomes apparent that cells displaying stained nuclei exhibit minimal Etho presence at elevated concentrations. The primary matrix structure consists of nanoparticles; the limited surface area and potential instability might have had a detrimental impact on cell adhesion. This observation can also be attributed to the surface energy-reducing properties of Etho within BFO nanoparticles. Nanoparticles possessing a high energy level on their uncoated surfaces tend to exhibit strong adsorption onto cell membranes through non-specific interactions, consequently reducing their overall surface energy [39]. With regard to the surface energy of nanoparticles, it is commonly defined as the quantification of the energy associated with unsaturated bonds originating from the exposed surface of a material [40, 41]. A surface exhibiting a high free energy level can enhance cell adhesion and spreading, whereas a surface with low free energy can impede cellular activity [42–44].

## Conclusions

Epilepsy, a multifaceted and disabling neurological condition, afflicts millions globally, particularly impairing the quality of life in cases of uncontrolled seizures. The management of epilepsy involves a variety of drugs with diverse structures, each exerting its effects through distinct molecular mechanisms. In our study, all nanoparticles underwent assessments related to their morphology, thermal properties, and cytotoxicity. Additionally, the drug release characteristics of ethosuximide were investigated in a PBS solution. However, it was noted that as the concentration of Etho increased, the drug release duration increased up to 5 hours, suggesting a more controlled release profile. The MTT assay results indicate that Etho-reinforced nanoparticles created an optimal environment for the non-toxicity, proliferation, and long-term survival of SH-SY5Y cells across multiple time points. Furthermore, it’s noteworthy that the viability of SH-SY5Y cells reached an impressive 135% even after 7 days of incubation. These findings suggest that BFO nanoparticles loaded with ethosuximide hold significant promise not only for treating epilepsy but also for potential applications in neural regeneration.
